# Evaluation of Betatrophin, Chemerin, and Kisspeptin Levels in Acromegaly Patients

**DOI:** 10.2174/0118715303375180250312035323

**Published:** 2026-04-21

**Authors:** Dondu Aysegul Cayan, Senay Topsakal, Esin Avci

**Affiliations:** 1 Department of Endocrinology and Metabolism, Faculty of Medicine, Pamukkale University, Denizli, Türkiye;; 2 Department of Biochemistry, Faculty of Medicine, Pamukkale University, Denizli , Türkiye

**Keywords:** Acromegaly, GH, betatrophin, chemerin, kisspeptin, IGF-1

## Abstract

**Background:**

Acromegaly is a chronic disorder characterized by excessive growth hormone (GH) secretion from pituitary somatotroph cells, resulting in metabolic complications and increased morbidity. Elevated levels of GH and insulin-like growth factor 1 (IGF-1) contribute to various metabolic and morphological abnormalities. Betatrophin, produced in the liver and adipose tissue, plays a significant role in regulating glucose and lipid metabolism. Chemerin, an adipokine, influences insulin sensitivity, lipid metabolism, and inflammation. Additionally, kisspeptin, a neuropeptide, stimulates the secretion of luteinizing hormone (LH) and follicle-stimulating hormone (FSH) through the hypothalamic-pituitary-gonadal axis.

**Objective:**

This study aimed to investigate the levels of betatrophin, chemerin, and kisspeptin in acromegaly patients and their potential relationship with dyslipidemia and diabetes.

**Methods:**

This study included 40 patients diagnosed with acromegaly and 40 healthy controls. GH and IGF-1 levels were measured, and venous blood samples were analyzed for betatrophin, chemerin, and kisspeptin levels using ELISA.

**Results:**

The analysis revealed no significant difference in betatrophin levels between acromegaly patients and controls (*p*>0.05). However, significantly lower levels of chemerin and kisspeptin were observed in patients with acromegaly (*p*<0.001). Furthermore, a significant correlation was identified between IGF-1 and kisspeptin levels, indicating a potential pathway for future research in diagnostics and therapy.

**Conclusion:**

This study highlights the altered levels of chemerin and kisspeptin in acromegaly patients, suggesting their involvement in metabolic dysregulation associated with the condition. Further investigation into the correlation between IGF-1 and kisspeptin could provide insights into developing targeted therapeutic strategies.

## 1 INTRODUCTION

Acromegaly is a rare and serious endocrinopathy, typically caused by excessive secretion of growth hormone (GH) from a pituitary adenoma originating from somatotroph cells. The chronic and uncontrolled release of GH leads to abnormal growth of bones and soft tissues, characterized by distinctive facial features, enlargement of the extremities, joint disorders, cardiovascular complications, insulin resistance, type 2 diabetes (T2DM), and respiratory system abnormalities. Diagnosis is often delayed due to the subclinical nature of the disease and the slow sprogression of symptoms, complicating the treatment process. If left untreated, acromegaly poses a significant risk for increased mortality, primarily due to cardiovascular and metabolic complications. Therefore, early diagnosis and treatment are crucial for improving quality of life and long-term survival. The development of acromegaly-specific biomarkers and more sensitive diagnostic methods could provide significant advancements in clinical management. Typically diagnosed in the fourth or fifth decade of life, its annual incidence is 2 to 11 cases per million, with a prevalence of 28 to 137 cases per million [1-4].

Betatrophin is a 198-amino acid protein produced by white and brown adipose tissues as well as the liver. In recent years, it has gained attention for its role in metabolic processes, particularly in the regulation of fat and glucose metabolism [5]. Betatrophin increases blood triglyceride levels by inhibiting lipoprotein lipase, a key enzyme in fatty acid metabolism. Due to this characteristic, betatrophin is thought to be linked to conditions where lipid metabolism is disrupted, such as metabolic syndrome, diabetes, and cardiovascular diseases. Studies in animal models have shown that administering high doses of betatrophin significantly raises triglyceride levels, even in healthy individuals [6-22].

Chemerin, first identified in 1998, is a protein primarily classified as an adipokine and secreted by adipose tissue, the liver, and various other tissues. It plays crucial roles in regulating inflammation, energy metabolism, and immune response. The biological activities of chemerin are mediated through signaling pathways triggered by its binding to specific cell surface receptors, particularly the chemokine-like receptor 1 (CMKLR1). Studies have reported that chemerin levels are significantly elevated in conditions, such as obesity, metabolic syndrome, type 2 diabetes, cardiovascular diseases, and chronic inflammatory states. As a result, chemerin is considered both a biomarker and a potential therapeutic target for these pathological conditions. Its role in metabolic and inflammatory processes makes chemerin a significant area of research for understanding the pathophysiology of obesity-related diseases and developing novel therapeutic approaches [23-38].

Kisspeptin, first isolated from the placenta in 2001, is a neuropeptide known for its significant effects on the reproductive system. It plays a critical role in regulating the hypothalamic-pituitary-gonadal (HPG) axis by stimulating the release of gonadotropin-releasing hormone (GnRH), which, in turn, increases the secretion of gonadotropins. These effects are mediated through a specific G-protein-coupled receptor known as GPR54. Beyond its central role in reproductive function, kisspeptin has garnered increasing interest due to its involvement in energy metabolism, pubertal development, cancer biology, and behavior. Additionally, studies have demonstrated that kisspeptin can exert both stimulatory and inhibitory effects on growth hormone (GH) secretion. Its diverse roles make kisspeptin a key target of research in the fields of endocrinology and reproductive biology. Research indicates that it stimulates GH secretion in sheep by activating the GPR54 receptor in pituitary cells. In contrast, endogenous kisspeptin has been shown to inhibit GH release through the same receptor [39, 40].

This study aims to identify the intricate relationship between kisspeptin, chemerin, and betatrophin in patients with acromegaly who are predisposed to dyslipidemia and diabetes, with the goal of identifying innovative, specific and highly sensitive biomarkers that could revolutionize the diagnosis and clinical management of this complex condition. By uncovering these critical biomarkers, we hope to pave the way for improved patient outcomes and more targeted therapeutic strategies in acromegaly care.

## 2 MATERIALS AND METHODS

### 2.1 Subjects and Study Design

This study was designed as a single-center, multidisciplinary, prospective clinical investigation. On May 21^st^, 2019, the Pamukkale University Clinical Research Ethics Committee granted the study ethical permission, with approval number 60116787-020/35532. Informed consent was obtained from all patients.

Based on previous studies, with an effect size of d=0.565, a type 1 error rate (α) of 0.05, and a study power (β) of 0.80, a sample size of 40 individuals per group was calculated using power analysis. In our study, a total of 40 patients diagnosed with acromegaly, aged between 18 and 70 years, who visited Pamukkale University Hospital's Endocrinology and Metabolic Diseases Outpatient Clinic, were selected. Additionally, 40 healthy individuals aged between 18 and 70 years, without a diagnosis of acromegaly, were included in the control group. Individuals under 18 years old or over 70 years old, those with a history of chemotherapy or radiotherapy, the presence of malignancy, serious systemic disorders, such as chronic liver and kidney diseases, and active smokers were excluded from both groups.

### 2.2 Biochemical Analysis

Serum levels of betatrophin, chemerin, and kisspeptin were investigated in both groups, along with GH and IGF-1 levels. Both the patient and control groups had an 8–12 hour overnight fast before having blood samples taken. The materials were transferred into 5-milliliter vacuum tubes together with a clot activator and yellow-capped gel. The samples were centrifuged for 10 minutes at 3500 rpm after being at room temperature for around 15 minutes. The levels of GH and IGF-1 were quickly determined by analyzing the resultant serum samples. In addition, three aliquots of serum samples were divided and kept at -80°C until the evaluation of kisspeptin, chemerin, and betatrophin levels. Chemiluminescent immunometric assays using the Immulite 2000 device were carried out to process serum samples for the determination of GH and IGF-1. The L2KGH kit was utilized for GH analysis, while the L2KGF2 kit was employed for IGF-1 analysis.

### 2.3 ELISA Method

For the measurement of human chemerin, betatrophin, and kiss-1 levels, commercial kits from AndyGene (Beijing Andy Huatai Technology Co., Ltd, Beijing, China) were utilized. All samples and kits were room temperature equilibrated before analysis. Standard and sample solutions were pipetted into microplate wells, following the instructions provided with the kits. The samples were then colorized based on test concentrations according to the specified protocol. The absorbance values of the wells were then measured using a Biotek Elx800 Microplate Reader (BioTek Instruments Inc., USA) at 450 nanometers (nm). Concentrations were calculated using the Gen5 data analysis program. The resulting values for GH, IGF-1, kisspeptin, chemerin, and betatrophin levels are expressed in ng/l units. All the tests were repeated three times for all patients. Additionally, routine biochemical serum parameters of all participants were also evaluated.

### 2.4 Statistical Analysis

The statistical package for social sciences, or SPSS, version 22.0, was used to analyze the data. The data were summarized using descriptive statistics; continuous variables were represented as the arithmetic mean (X̄) and standard deviation (SD), and categorical variables were reported as the number (n) and percentage (%). In order to determine whether the data had a normal distribution, tests for normality, such as the Shapiro-Wilk and Kolmogorov-Smirnov tests, were performed. Mann-Whitney U and Kruskal-Wallis tests were used to compare the variations in biochemical parameters between the patient and control groups because of the non-normal distribution of the data. Yates' Chi-square test was carried out to evaluate group differences for categorical variables. To examine the connections between continuous variables, Spearman’s correlation coefficient was computed. The significance threshold of *p*<0.05 was employed.

## 3 RESULTS

### 3.1 Anthropometric Results

Our study comprised 80 individuals, evenly divided into an acromegalic patient group and a control group, each consisting of 40 individuals. Gender distribution was similar in both groups, with 19 males (47.5%) and 21 females (52.5%) in each. Statistical analysis indicated no significant difference in gender distribution between the patient and control groups (*p* > 0.05).

The average age of the patient group was 52.28±10.84 years, ranging from 28 to 65 years old. Similarly, the mean age of the control group was 52.3±10.84 years, ranging from 28 to 67 years old. The mean ages of the study and control groups did not significantly differ from one another, according to statistical analysis (*p* > 0.05).

### 3.2 Biochemical Results

Upon examination of our study group, consisting of patients diagnosed with acromegaly, mean serum betatrophin levels were 470.085 ± 386.357 ng/L, ranging from 121.299 ng/L to 1853.044 ng/L. In comparison, the mean serum betatrophin level in the control group was 645.164 ± 588.582 ng/L, with levels ranging from 86.886 ng/L to 2762.772 ng/L. The mean betatrophin levels of the patient and control groups did not differ statistically significantly, according to the statistical analysis (*p*>0.05).

Additionally, the mean serum chemerin level in the acromegaly patient group was 11.019 ± 10.431 ng/L, with levels ranging from 5.951 ng/L to 72.121 ng/L. Conversely, in the control group, the mean serum chemerin level was 18.545 ± 21.419 ng/L, ranging from 6.192 ng/L to 114.370 ng/L. The mean chemerin levels of the patient and control groups differed significantly (*p*<0.001), with individuals with acromegaly showing lower levels.

In addition, the mean serum kisspeptin level in our study group of individuals with acromegaly was 600.451 ± 169.779 ng/L, with a range of 263.073 ng/L to 1171.492 ng/L. In contrast, the mean serum kisspeptin level in the control group was 752.006 ± 170.559 ng/L, with levels ranging from 518.519 ng/L to 1271.825 ng/L. The mean levels of kisspeptin in the patient and control groups differed significantly (*p*<0.001), with lower levels seen in acromegaly patients, according to statistical analysis. The comparative mean values of the analyzed three biomarkers, as well as GH and IGF-1 levels for the patient and control groups, are provided in Fig. (**1**). Table **1** presents the relationship between GH and IGF-1 and betatrophin, chemerin, and kisspeptin in the acromegaly group. Fig. (**2**) shows the statistically substantial connection (*p*<0.01) between groups and kisspeptin with IGF1. Blood glucose, creatinine, and lipid profiles (total cholesterol, triglyceride, low-density lipoprotein (LDH), and high-density lipoprotein (HDH), are shown in Fig. (**3**).

## 4 DISCUSSION

In this study, we investigated betatrophin, chemerin, and kisspeptin levels in acromegaly patients. While no significant difference was found in betatrophin levels, there were statistically significant decreases in chemerin and kisspeptin levels. Additionally, a statistically significant correlation between kisspeptin and IGF-1 levels was identified in the acromegaly group. These findings suggest that examining chemerin and kisspeptin may provide important insights into the early diagnosis and possibly the treatment of acromegaly.

Acromegaly is a complex disorder caused by excessive secretion of GH from the pituitary gland, regulated by the HPG axis [41-44]. Within this axis, kisspeptin plays a pivotal role by stimulating the release of GnRH, which, in turn, modulates LH and FSH levels [45-52]. Studies suggest that kisspeptin may also influence GH secretion in acromegaly patients, with some research indicating that kisspeptin levels might be altered in these patients and could be linked to metabolic and reproductive disorders [53-56]. Kisspeptin, primarily produced in the hypothalamus, is well-known for activating GnRH neurons, thereby influencing energy balance and reproductive function [45]. Animal studies have highlighted the role of kisspeptin in growth hormone (GH) secretion. For instance, Han *et al*. demonstrated that administering kisspeptin to juvenile and prepubertal mice induced greater depolarization of GnRH neurons in the prepubertal group, suggesting its involvement in pubertal development [56]. Similarly, Foradori *et al*. found that administering intracerebroventricular kisspeptin-10 to lambs after fasting resulted in a significant rise in plasma GH levels [54]. In another study, Smith *et al*. reported that intraventricular administration of kisspeptin antagonists to sheep led to a notable increase in GH levels while reducing luteinizing hormone (LH) levels without affecting cortisol or prolactin levels [55]. In our study, we observed significantly lower kisspeptin levels in acromegaly patients compared to controls. Furthermore, a statistically significant correlation between kisspeptin and IGF-1 levels was identified in the acromegaly group. These findings suggest that kisspeptin interacts with IGF-1 levels and that this connection may play an important role in the pathophysiology of acromegaly. However, further research is needed to better understand the mechanisms underlying this relationship.

Betatrophin, a protein primarily synthesized in the liver and adipose tissue, plays an essential role in glucose and lipid metabolism. Previous studies indicated that betatrophin levels may fluctuate in acromegaly patients, with potential links to metabolic syndrome, insulin resistance, and dyslipidemia [57]. However, our study found no significant difference in betatrophin levels between acromegaly patients and controls.

Regarding betatrophin, this protein has been shown to raise triglyceride levels by inhibiting lipoprotein lipase (LPL) and altering very low-density lipoprotein (VLDL) secretion from the liver [58]. Research on betatrophin levels in obesity and T2DM has yielded contradictory findings. While some studies revealed no significant difference, others observed 1.8-fold increased levels of betatrophin in T2DM and obesity [59, 60]. When compared to lean and non-diabetic people, Gomez-Ambrosi *et al*. found that patients with T2DM and obesity had reduced serum betatrophin levels. Additionally, they found that there was a positive link with HDL cholesterol and a negative correlation with serum betatrophin levels and body mass index (BMI) [61]. Patients with obstructive sleep apnea had higher betatrophin levels than controls in research conducted by Sertogullarindan *et al*. [62]. Nonetheless, there was no discernible variation in blood betatrophin levels between the control and acromegaly groups in our investigation. This result suggests that betatrophin does not play a significant role in the pathogenesis of acromegaly.

Acromegaly is also associated with significant metabolic disturbances, including insulin resistance and chronic inflammation, driven by elevated GH and IGF-1 levels [63]. Chemerin, a protein implicated in regulating metabolic functions, has emerged as a key marker for metabolic disorders, such as metabolic syndrome, obesity, and cardiovascular diseases, often observed in acromegaly patients [64]. This situation indicates that chemerin may play a crucial role in understanding metabolic disorders associated with acromegaly and could serve as a potential biomarker for this condition, which led to the planning of this study. However, the role of chemerin and the changes in its levels in acromegaly are not yet fully understood, including how these changes relate to the metabolic dysfunction associated with the disease. Nevertheless, our study found that chemerin levels were significantly lower in patients with acromegaly.

Chemerin plays a pivotal role in regulating processes, such as inflammation, adipogenesis, and insulin sensitivity. Inflammatory joint conditions, including hypertrophic arthropathy, are common in acromegaly, and chemerin has been associated with these inflammatory disorders. Several studies have demonstrated elevated chemerin levels in conditions like metabolic syndrome and rheumatic diseases, emphasizing its role in chronic inflammation [65-68]. For instance, Amorim *et al*. identified a link between the KISS1 c.-145delA (rs5780218) promoter gene polymorphism and the prevalence of somatotropinoma in acromegaly patients [69]. Research by Bozaoglu *et al*. also found higher chemerin gene expression in adipose tissue of individuals with obesity and T2DM [70]. Furthermore, chemerin levels have been reported to be elevated in the synovial fluid of patients with knee and temporomandibular joint osteoarthritis [71, 72]. Numerous studies have explored the involvement of chemerin in chronic inflammatory conditions, such as diabetes, cardiovascular disease, and rheumatic diseases, further underscoring its potential role in these pathologies [37, 73-76]. A study by Wu examining adiponectin, chemerin, and vascular endothelial growth factor (VEGF) in relation to epicardial fat volume in patients with coronary artery disease found that higher chemerin levels were associated with cardiovascular risk in those with metabolic syndrome [77].

Reverchon *et al*. also demonstrated that chemerin and its receptor CMKLR1, when isolated from human ovarian granulosa cells, inhibit steroidogenesis and cell proliferation by reducing IGF-1 receptor tyrosine phosphorylation when granulosa cell cultures are exposed to recombinant chemerin [65]. These findings highlight the significant role of chemerin in hypothalamic-pituitary function. In line with this, our study found that acromegaly patients had significantly lower serum chemerin levels compared to controls, suggesting a possible role for chemerin as a biomarker in acromegaly.

GH and IGF-1 dysregulation in acromegaly contributes to metabolic disruptions, such as increased gluconeogenesis, glycogenolysis, and impaired glucose tolerance, often leading to insulin resistance and dyslipidemia. It is well-established that these patients may present with decreased HDL cholesterol and reduced hepatic lipoprotein lipase activity [1]. In our study, we observed that while there were no significant differences in LDL, HDL, or total cholesterol levels between acromegaly patients and controls, triglyceride and fasting glucose levels were significantly elevated.

This study has several limitations. Due to acromegaly being a rare disease, the study was conducted with a sample size of only 40 patients. Future studies should evaluate the patients' broader anthropometric, hormonal, biochemical outcomes, and imaging results from a more comprehensive perspective.

The innovation of this research lies in the novel investigation of the correlations between betatrophin, chemerin, and kisspeptin with GH and IGF-1 levels in patients with acromegaly. While previous studies explored individual biomarkers in acromegaly, this is the first study to evaluate the combined roles of these specific serum factors in the context of acromegaly, a condition predisposed to dyslipidemia and diabetes. Additionally, this study explored the potential implications of these factors in understanding the metabolic disturbances associated with acromegaly, which could pave the way for more targeted therapeutic approaches in managing this rare disease.

Our study reveals a complex interaction between acromegaly and the levels of kisspeptin, betatrophin, and chemerin. Notably, while betatrophin levels did not differ significantly between acromegaly patients and the control group, both kisspeptin and chemerin levels were significantly lower in acromegaly patients. This result particularly suggests that kisspeptin and chemerin may play significant roles in the pathogenesis of acromegaly.

## CONCLUSION

In conclusion, this study underscores the complex interplay between kisspeptin, betatrophin, and chemerin in acromegaly, particularly regarding metabolic and hormonal imbalances. While kisspeptin and chemerin levels were significantly reduced in acromegaly patients, betatrophin levels remained unchanged, indicating the need for further investigation into the distinct roles of these molecules in acromegaly pathophysiology. Our findings highlight the critical function of kisspeptin within the hypothalamic-pituitary axis, especially its influence on GH secretion. Although GH levels are typically elevated in acromegaly, the underlying mechanisms are not fully understood. This study examined the relationships between serum kisspeptin, chemerin, and betatrophin levels in individuals with metabolic disturbances and acromegaly, as significantly lower levels of kisspeptin and chemerin were found in patients compared to controls, while betatrophin levels showed no difference. A significant correlation between kisspeptin and IGF-1 levels was also observed in the patient group. These results suggest that kisspeptin and chemerin may serve as promising biomarkers for understanding the metabolic and hormonal disruptions in acromegaly. However, further research is needed to clarify their roles and develop targeted therapeutic strategies for this complex condition.

This study is the first to investigate serum levels of kisspeptin, betatrophin, and chemerin in a relatively small group of patients diagnosed with acromegaly, a relatively rare endocrinopathy. Analyzing data from a larger number of patients could yield clearer results.

## Figures and Tables

**Fig. (1):**
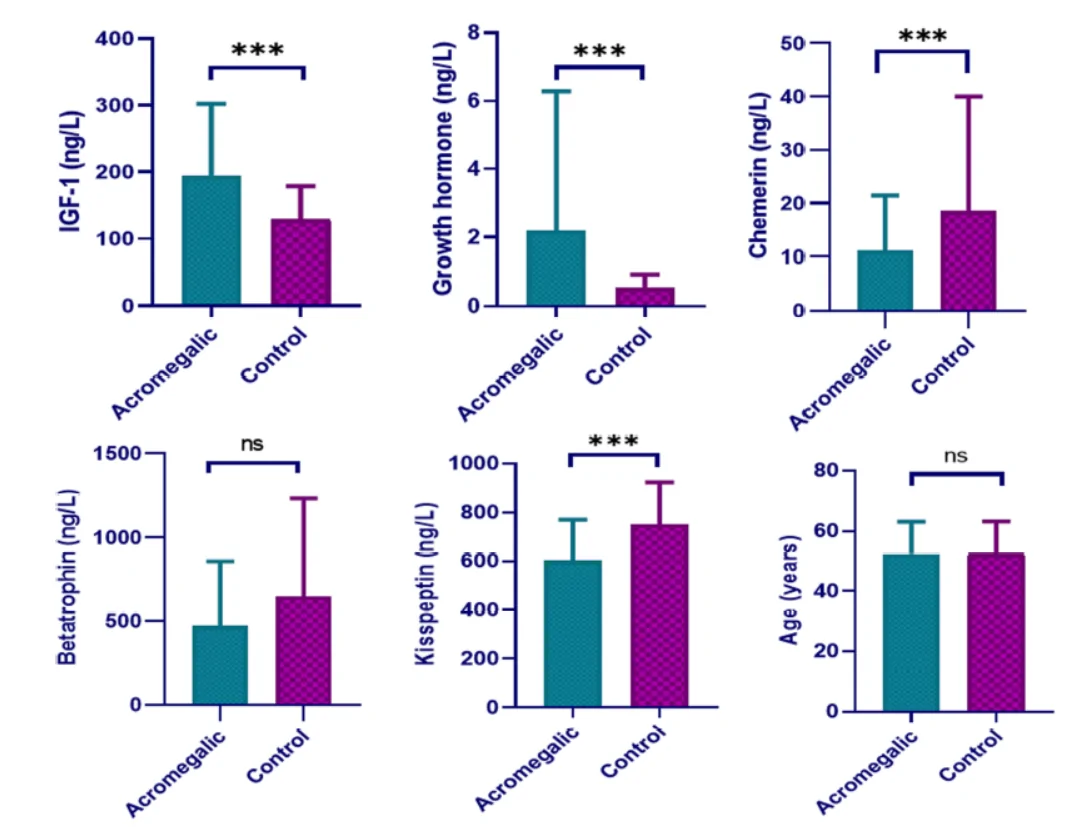
Graphical representation of hormonal levels and ages of acromegalic patients and controls. This figure shows the comparison of hormonal levels (IGF-1, GH, chemerin, betatrophin, and kisspeptin) and ages between acromegalic patients and the healthy control group. ns: not significant, ***: *p*>0.001.

**Fig. (2):**
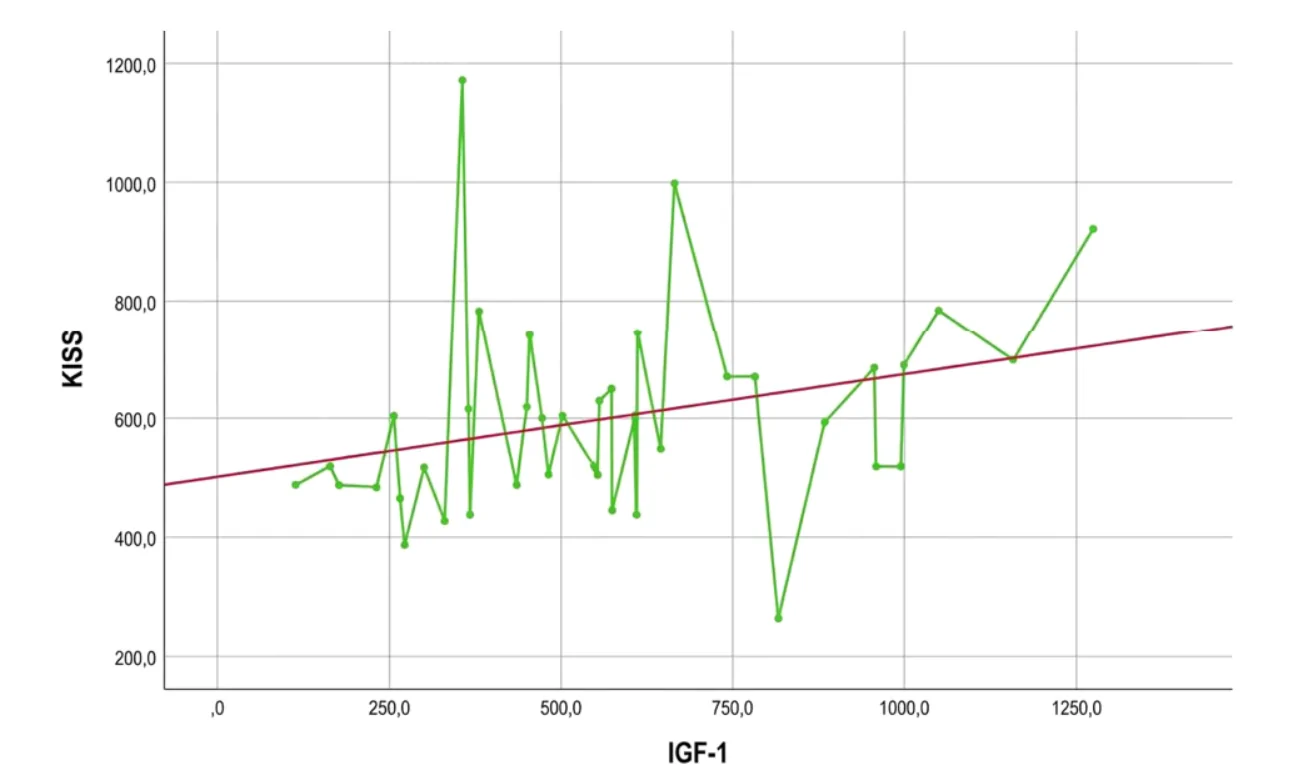
Relationship between kisspeptin and IGF-1 in the acromegaly study group (significant at the 0.01 level of correlation). This figure illustrates the correlation between kisspeptin levels and IGF-1 levels in patients with acromegaly. A statistically significant relationship at the 0.01 level suggests that as kisspeptin levels increase or decrease, there is a corresponding change in IGF-1 levels within the acromegaly group.

**Fig. (3):**
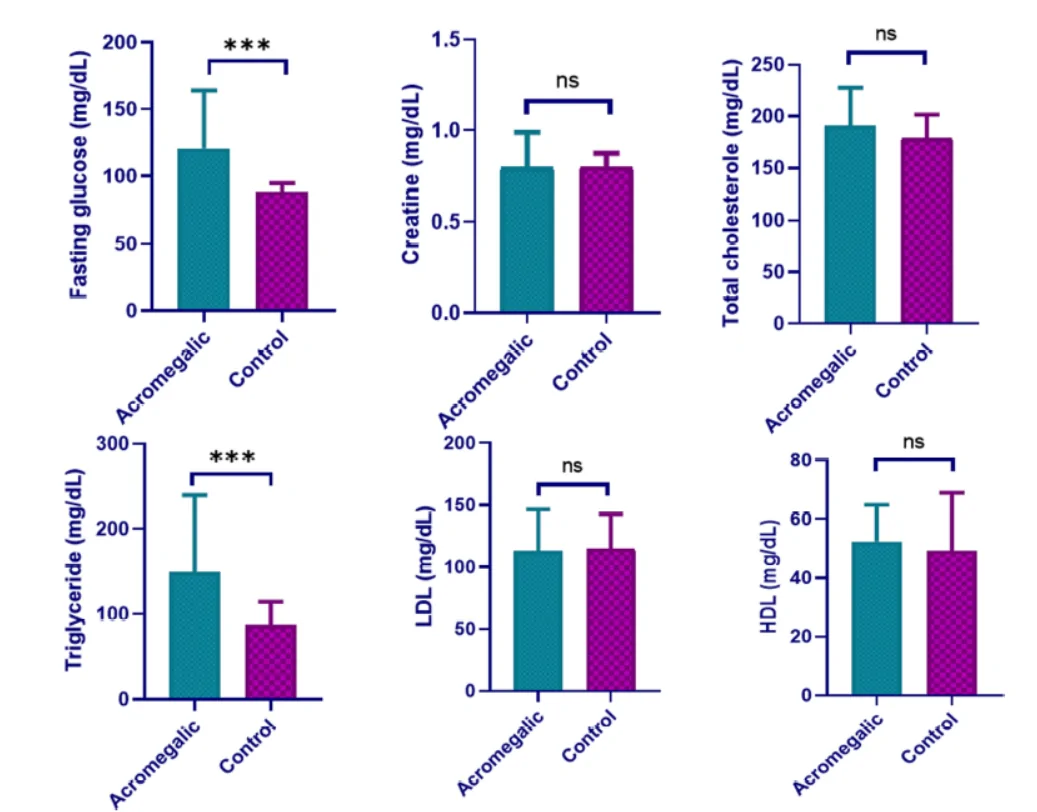
Comparison of fasting glucose, creatinine, and blood lipid profiles (such as total cholesterol, triglyceride, LDL, and HDL) between acromegalic patients and controls. The statistical results indicate that there are significant differences in these parameters between the two groups. ns: not significant, ***: *p*<0.001.

**Table 1:** Correlation of betatrophin, chemerin, and kisspeptin with GH and IGF-1.

-	-	Betatrophin	Chemerin	Kisspeptin
GH (ng/L)	r	-0.072	-0.294	0.089
	p	0.659	0.066	0.584
IGF1(ng/L)	r	0.147	-0.017	0.408**
	p	0.365	0.918	**0.009**

**Note:** Pearson correlation, **: Correlation is significant at the 0.01 level, (-): Negative correlation, r: correlation coefficient, p: significance level.

## Data Availability

The data and supportive information are available within the article.

## LIST OF ABBREVIATIONS

CMKLR1: Chemokine-like Receptor 1
LDL: Low-density Lipoprotein
HDL: High-density Lipoprotein
VLDL: Very low-density Lipoprotein
IGF-1: Insulin-like Growth Factor 1
GH: Growth Hormone
T2DM: Type 2 Diabetes Mellitus
LPL: Lipoprotein Lipase
HPG: hypothalamic-pituitary-gonadal
GnRH: Gonadotropin-releasing Hormone
GPR: G-protein-coupled Receptor

## STANDARDS OF REPORTING

STROBE guidelines were followed.
